# Information and authority: research on the mechanism of offspring's health information avoidance behavior

**DOI:** 10.3389/fpubh.2025.1516225

**Published:** 2025-07-07

**Authors:** Qiong Dang, Weiying Lin, Yifei Li

**Affiliations:** ^1^Center for Digitalized Culture and Media, University of Electronic Science and Technology of China, Chengdu, China; ^2^School of Journalism and Communication, Guangxi University, Nanning, China; ^3^Chengdu Star Era Aerospace Technology Co., Ltd., Chengdu, China

**Keywords:** family communication patterns, conversation orientation, conformity orientation, information acceptance model, health information avoidance

## Abstract

The health information shared by parents with their offspring, while originating from a place of care, has in practice led to resistance from the offspring. This has become a pressing issue in the field of health communication in contemporary China that requires urgent attention and resolution. Therefore, the study aims to explore how information factors and family factors together affect offspring's avoidance of health information shared by parents, within the context of the unique characteristics of social and family relationships in China. This study, based on the theoretical framework of the information acceptance model (IAM) and family communication patterns (FCPs), takes offspring under 30 years old as the research object, analyzes 1,505 valid questionnaires using SEM. The results revealed that within FCP, conversation orientation negatively impacts offspring's health information avoidance (HIA) behavior, while conformity orientation positively influences this behavior. An important finding is that parental authority and discourse power are the most significant determinants of offspring's HIA behavior. Additionally, the quality of health information and source credibility each positively affect offspring's perceived usefulness of the health information. Moreover, both perceived usefulness and attitude have a significant association with HIA behavior, with attitude mediating the relationship between perceived usefulness and HIA behavior. An interesting finding is that offspring's emotional responses and subjective attitudes play a critical role in the health information decision-making process. This study addresses the lack of focus on family communication patterns and information systems in health communication research and provides new insights for future studies.

## 1 Introduction

The family is a crucial context for health communication. As both a socialization agent and an emotional support system, the family plays a foundational role in shaping individuals' health beliefs, behaviors, and communication patterns (1, 2). In particular, within the domain of public health, family-based health communication is increasingly recognized as a primary site for informal health information exchange and behavior modeling (3). Traditionally, in the Chinese family setting, health communication primarily involves the transmission of health knowledge and information sharing from parents to offspring (4). Within the hierarchical family structure and the cultural norms of traditional Chinese filial piety, individuals are often expected to obey their elders and refrain from questioning or challenging their authority (5). As an ancient Chinese saying, “A filial son nourishes his parents by bringing joy to their hearts and not going against their wishes.” This cultural norm may influence how younger family members interpret and respond to health information shared by their parents, often perceiving such advice as authoritative and deserving of respect. Therefore, offspring must carefully consider how to avoid intergenerational conflict when faced with health information shared by their parents. When faced with overwhelming health rumors in family WeChat groups or pseudo-health and pseudo-science articles forwarded by parents, offspring often find themselves in an awkward situation (3). However, even if the health information shared by parents is scientifically valid and useful, offspring may still tend to avoid it (6). This scenario reflects relational tension and role conflict within the family. The younger generation often perceives the forwarded content as lacking credibility but chooses to remain silent or disengaged to preserve family harmony. Such avoidance serves as a relationship-driven coping mechanism shaped by intergenerational dynamics and cultural expectations, particularly within collectivist societies (7–9), and is central to understanding Health Information Avoidance (HIA) in family contexts. They are aware that they should not remain silent, yet hesitate to refute the information in order to maintain family harmony. In recent years, driven by factors such as social progress, educational advancement, legal protections, and shifts in family values, modern families are evolving toward a more democratic, equal, and respectful approach to individual rights (10). While these changes promote autonomy and open dialogue, they may also reduce the traditional deference to parental authority, making younger generations more likely to question or resist unsolicited advice (9, 11, 12)—including health-related messages—from their parents. An increasing emphasis on individual voice and critical thinking may intensify intergenerational friction. This tension becomes more pronounced when older family members expect their views to be accepted without question (13). As issues related to family and health become increasingly prominent in Chinese society, offspring's avoidance of health information shared by their parents has become a particularly worrying reality. This phenomenon reflects potential intergenerational conflicts and communication barriers within modern families during the exchange of health information (14).

In the Chinese context, the concern traditionally conveyed through parental health information sharing has gradually transformed into a burden of information for the younger generation. Chen and Gan (4) pointed out that offspring increasingly assumes the role of “expectation violators” in the process of health information sharing by older adults. They are less likely to provide feedback to their parents on how they feel about the shared information and more frequently point out errors in the information shared by older adults. Yao and Shen ((15), p. 83) found that communication between the offspring and parental generations often exhibits a pattern where parents send information one-way as a form of emotional connection, but the younger generation tends to respond with a “read without reply” approach, refusing to engage in further communication. Despite the fact that parents do not frequently forward or share online information with their offspring, the younger generation still tends to develop feelings of resentment and fatigue (16). The current study shifts focus away from parental motivations and emphasizes offspring's cognitive and emotional responses to parental health information sharing. Specifically, it examines how relational tension, perceived credibility, and communication patterns shape the tendency among offspring to avoid engagement with health information received from their parents.

While previous research has primarily examined the motivations behind parental health information sharing on social media, less attention has been paid to how offspring perceive and respond to such behavior. In many cases, younger individuals tend to view health information forwarded by their parents—especially through platforms like WeChat—as outdated, exaggerated, or irrelevant, which contributes to feelings of annoyance, fatigue, or emotional resistance (17, 18). Several studies have suggested that this form of avoidance is influenced by relational dynamics, digital literacy gaps, and intergenerational value differences (19). Specifically, younger individuals may refrain from confronting misinformation not only to avoid conflict but also due to perceived futility or fear of damaging familial relationships (20, 21). Despite growing interest in health information behaviors, research has rarely explored the psychological and communicative mechanisms that lead offspring to avoid health information in intergenerational family settings—particularly in the Chinese cultural context where filial piety and face-saving norms further complicate such interactions. HIA refers to individuals' deliberate efforts to avoid, ignore, or dismiss health-related information that may cause emotional discomfort, cognitive dissonance, or interpersonal tension (8, 22). In family contexts, offspring may engage in HIA when faced with health suggestions from their parents, even when the information is well-intentioned or scientifically accurate. This behavior is a multifaceted phenomenon influenced by factors such as information credibility, parent–child communication dynamics, perceived relational obligations, and cultural expectations.

Therefore, the study aims to focus on the phenomenon of offspring avoiding health information shared by their parents within the family context, set against the backdrop of media technology transformation. This research specifically focuses on Chinese families, where unique cultural norms such as filial piety and respect for familial authority are deeply embedded in intergenerational communication (11). Filial piety, a core concept in Confucianism, emphasizes obedience, respect, and care for one's parents and elders, often discouraging open disagreement or confrontation (23–25). These deeply rooted norms influence how health information is exchanged and negotiated within families, especially when younger family members are expected to prioritize relational harmony over personal autonomy.

In this context, challenging or rejecting health advice from parents is not merely a cognitive act but also a cultural one, potentially perceived as disrespect or disobedience. Moreover, modern Chinese families are undergoing a transformation from traditional hierarchical structures toward more egalitarian and dialogic communication styles, which may heighten generational tensions when it comes to sharing or contesting health information. This tension has not been sufficiently explored in existing research. Despite the increasing relevance of health information behaviors in the digital age, few studies have applied the Information Adoption Model (IAM) and Family Communication Patterns (FCPs) framework to understand HIA within the Chinese intergenerational context. This study addresses that gap by integrating these frameworks to explain how informational and relational factors co-shape avoidance behaviors. Furthermore, this study draws upon recent Chinese and international research that highlights the impact of digital platforms (e.g., WeChat) on intergenerational health communication, emotional fatigue, and silent resistance among youth [e.g., (4, 15, 17)].

## 2 Theoretical foundation

### 2.1 Health information sharing and avoidance in the family context

In the context of Chinese society, offspring's avoidance of health information shared by their parents reflects both cognitive evaluation-central to the Information Adoption Model (IAM)-and relational regulation, as explained by Family Communication Patterns (FCPs). A single theoretical framework is insufficient to fully explain the complexity of this behavior. Therefore, this study integrates the Information Adoption Model (IAM) and the Family Communication Patterns (FCPs) Theory to construct a dual-pathway framework that explains HIA from both informational and relational perspectives. The IAM emphasizes that individuals decide whether to adopt or reject information based on their evaluation of its quality and the credibility of the source (26–28). Offspring in contemporary Chinese families often question the scientific validity or usefulness of health information forwarded by their parents, regardless of the medium through which it is shared. This skepticism is rooted in generational differences in health literacy and perceived credibility of the information source (9). As a result, they may engage in health information avoidance, reflecting a rational, content-based rejection shaped by relational and informational dynamics.

However, the IAM alone cannot explain why offspring who recognize flawed information often choose not to directly correct or confront their parents, but instead remain silent or respond passively. This behavior is deeply shaped by the relational norms within Chinese families, where values such as filial piety and deference to elders play a central role in shaping intergenerational communication (5, 29). The FCPs theory offers a structural approach to understanding these dynamics, distinguishing between “conformity-oriented” and “conversation-oriented” communication styles (30, 31). In conformity-oriented families, offspring are expected to comply with parental guidance and minimize dissent. As a result, they may cognitively reject the health information but behaviorally avoid direct resistance—leading to HIA as a relational coping mechanism. In contrast, conversation-oriented families allow for more open discussion, potentially reducing avoidance behaviors. By integrating IAM and FCPs, this study seeks to capture the dual mechanisms—cognitive rejection and relational suppression—that jointly drive HIA. This theoretical integration enriches the understanding of health information behavior and enhances the applicability of the model within the Chinese socio-cultural context.

### 2.2 Information acceptance model

Sussman and Siegal (26) proposed the Information Acceptance Model (IAM), which explains how individuals adopt or reject information based on their evaluations of information quality, source credibility, and perceived usefulness. Source credibility refers to the extent to which individuals believe the information source is reliable and trustworthy (32). Information quality involves subjective judgments about the completeness, accuracy, and relevance of information in a specific context (33). Perceived usefulness refers to people's recognition of the actual value of the health information provided (34). These three dimensions together determine whether information is considered worth accepting or engaging with. To date, IAM has been widely employed to explore information behaviors, such as the acceptance, processing, and rejection of information (35–37). While IAM was initially developed in organizational and digital environments, its theoretical logic—rooted in dual-process models of information processing—has broad applicability to interpersonal and health-related contexts, including family communication (38).

In the context of intergenerational family communication, particularly between parents and offspring, information acceptance is not a purely rational act but a complex interplay between cognitive assessment and relational dynamics. Offspring do not passively receive health information from parents; rather, they engage in active evaluation—assessing not only the factual reliability of the content (information quality), but also the credibility of the source (i.e., the parent), and the personal relevance or utility of the message (perceived usefulness) (39). While these three dimensions are core constructs of the IAM model, their relevance alone does not justify its use in family settings. The IAM framework is particularly appropriate for analyzing HIA behavior in intergenerational family contexts because it accounts for how individuals cognitively process and evaluate information based on perceived credibility and relevance. In families, where emotional bonds, hierarchical dynamics, and cultural values like filial piety influence information reception, IAM provides a useful lens to understand why offspring may choose to accept or avoid health advice shared by their parents. In Chinese families, where communication is often shaped by filial piety and respect for authority, the source (parent) holds a dual role: a transmitter of information and a figure of relational obligation. This amplifies the role of source credibility and introduces emotional complexity into the cognitive decision to accept or avoid information.

In addition, IAM has been enriched by the inclusion of new explanatory variables. One such variable is “attitude toward information,” which has gained increasing attention in recent studies. Attitude refers to an individual's evaluative disposition—positive or negative—toward the information being received, which encompasses both cognitive judgment and emotional reaction (40). In the context of HIA, offspring may cognitively recognize the value of health information shared by parents, yet still avoid engaging with it due to a negative emotional attitude—such as perceiving the information as annoying, intrusive, or threatening to autonomy. This disconnect between perceived usefulness and behavioral response highlights the mediating role of attitude in decision-making processes. Several studies support the inclusion of attitude in extended IAM frameworks. Park (41) found that individuals' attitudes—whether they view information as annoying, emotionally burdensome, or personally relevant—significantly influence their likelihood of accepting or avoiding it. Le (42) further demonstrated that negative attitudes toward health messages were strong predictors of information avoidance behavior. Given that HIA behavior often occurs in emotionally charged interpersonal contexts, such as parent-offspring communication, the inclusion of attitude provides a more nuanced understanding of the decision to engage with or avoid information. These findings suggest that without considering attitude, the IAM framework may underestimate the emotional and motivational aspects of information processing—particularly in family environments where relational dynamics play a central role. Therefore, this study incorporates “attitude toward information” as a fourth key variable in the IAM framework. Therefore, this study builds upon the IAM framework by incorporating four key variables: information quality, source credibility, perceived usefulness, and attitude. This extended model not only identifies critical antecedents of offspring's HIA behavior but also enhances our understanding of the complex emotional and cognitive processes involved in family health information exchange.

### 2.3 Family communication patterns: conversation orientation and conformity orientation

The FCPs model was proposed by McLeod and Chaffee (43) and refers to the ways in which family members, particularly parents and offspring, interact and communicate with each other. The FCPs model was originally developed to examine how family members—especially parents and children—communicate in the context of media use and political socialization. Ritchie and Fitzpatrick (30) further enriched and deepened the FCPs model by dividing it into two dimensions: conversation orientation and conformity orientation. Conversation orientation refers to the extent to which family members share their views, feelings, and beliefs with one another. Families with a high Conversation orientation encourage extensive and unrestricted communication among all members, where family members are relatively equal and the frequency of communication is high (3). Conformity orientation refers to the clear hierarchical relationships among family members, where emphasis is placed on the obedience of offspring to parents (44). In a family with a high conformity orientation, there is limited open discussion on topics, and the frequency of interaction is relatively low (45). Over time, this model has been extended to other interpersonal communication domains, including health communication within families. A large number of empirical studies have shown that the FCPs significantly affects family members' behaviors in obtaining, sharing, and adopting health information (46–49). For instance, Hovick et al. (48) demonstrated that a family communication model that emphasizes consistency and authority may inhibit individuals' willingness to actively seek key information, while a conversation-oriented pattern that encourages free expression and open discussion facilitates smoother information exchange and communication.

In addition, the applicability of FCPs to the Chinese intergenerational context is evident in the model's emphasis on authority and dialogue. These dimensions align well with traditional Chinese family communication norms shaped by Confucian values such as filial piety and respect for hierarchy. In families where obedience to elders is culturally expected, conformity-oriented communication may dominate, while in more modernized families, conversation orientation has been gradually increasing (3, 50). Several empirical studies in East Asia have confirmed the cultural relevance of FCPs in parent-offspring interactions (51–53). For example, Kang et al. (51) and Xie (53) applied FCPs in Korean and Chinese family settings respectively, demonstrating that family communication orientation significantly affects adolescents' openness and information engagement. Moreover, Gong et al. (49) who found that in conversation-oriented Chinese families, middle-aged and older adults expressed less negative emotions in response to infectious disease prevention and control. These studies suggest that FCPs demonstrates strong explanatory power in collectivist and hierarchy-oriented social contexts.

Therefore, FCPs is not only a crucial theoretical tool for understanding the exchange of health information within the family, but also a significant factor in predicting and explaining the health behaviors of family members. This study took the conversation orientation and conformity orientation of FCPs as two core variables at the family factor level to reveal how different FCPs affect offspring's behaviors toward health information shared by their parents.

## 3 Research hypotheses

### 3.1 Relationships between conversation orientation, conformity orientation, and offspring's HIA behavior

The two dimensions of FCPs—conversation orientation and conformity orientation—have been extensively applied in studies examining health behavior and intergenerational information exchange (49, 54). Conversation orientation encourages open dialogue, mutual understanding, and emotional sharing among family members. In such environments, offspring are more likely to express their opinions and critically engage with health-related topics. For example, Moss et al. (55) found that individuals from high conversation-oriented homes were significantly more willing to discuss COVID-19 vaccination with their parents. Similarly, Scheinfeld (54) demonstrated that conversation orientation positively mediated both the willingness to disclose health concerns to parents and to third parties. These findings indicate that a high level of conversation orientation fosters open, supportive dialogue, which in turn helps reduce communicative barriers and emotional resistance. As a result, it may significantly decrease the likelihood of HIA in family contexts. Conformity orientation, on the other hand, emphasizes family hierarchy, obedience, and the suppression of dissent. In such families, offspring may avoid challenging or rejecting parental information to preserve relational harmony. Hesse and Rauscher (47) found that conformity orientation negatively influenced vaccine intentions. Moreover, Bakhtiari (56) showed that conformity-oriented communication styles predicted increased psychological distress and behavioral withdrawal among students, including anxiety, low self-esteem, and avoidance behavior. For example, Zhou et al. (6) found that individuals often avoid certain health-related topics with close family members to reduce anxiety and maintain relational harmony. This suggests that in conformity-oriented families, HIA may serve as a strategy for passive resistance or emotional protection. Based on the theoretical and empirical evidence above, the following hypotheses are proposed:

Hypotheses 1a (H1a) Conversation orientation negatively influences offspring's HIA behavior.
Hypotheses 1b (H1b) Conformity orientation positively influences offspring's HIA behavior.

### 3.2 Relationships between information quality, source credibility, and perceived usefulness

In the IAM, perceived usefulness is conceptualized as a key mediator in determining whether individuals will accept or avoid certain information (26). Information quality refers to the accuracy, clarity, and relevance of the content, while source credibility reflects the extent to which the sender is viewed as trustworthy and knowledgeable. Both of these factors shape how useful the information appears to the recipient.

High information quality enhances perceived usefulness by providing clear, reliable, and actionable content, thereby increasing the likelihood that users will find the information worth engaging with. Komendantova et al. (57) demonstrated this effect in their study on Instagram, showing that higher-quality information significantly increased users' willingness to adopt content related to renewable energy. In a health communication context, clear and evidence-based health messages are more likely to be viewed as beneficial, especially by younger individuals facing information overload. Source credibility, although more relational in nature, also plays a critical cognitive role. A message from a trusted source is more likely to be processed positively, especially in close relationships such as family. Prior research shows that when the sender is perceived as credible—emotionally invested, knowledgeable, or morally authoritative—the recipient is more inclined to consider the information useful (58, 59). This relationship is particularly important in intergenerational communication, where authority and emotional ties are deeply intertwined (9).

Hypotheses 2 (H2) The quality of health information shared by parents positively influences offspring's perceived usefulness of the information.
Hypotheses 3 (H3) The perceived credibility of parents as sources of health information positively influences offspring's perceived usefulness of the information.

### 3.3 Relationships between perceived usefulness, attitudes, and HIA

In the IAM framework, perceived usefulness refers to the extent to which individuals believe that health information is valuable, relevant, and beneficial to their decision-making (26). This perception not only increases the likelihood of accepting information but also influences the individual's emotional and cognitive attitude toward it. The link between perceived usefulness and attitude has been well-documented. Peng et al. (60) found that when patients perceive medical information as useful, they tend to form more favorable attitudes toward sharing it. Similarly, Tan et al. (61) demonstrated that usefulness of the system positively and significantly influences students' favorable attitudes toward hybrid learning. This suggests that the more useful the information is perceived to be, the more positively it is received on an attitudinal level. Attitude, in turn, plays a central role in the decision to engage with or avoid health information. According to behavioral theories such as the Theory of Planned Behavior (40), attitudes toward a behavior significantly predict behavioral intentions and actions. Zhang and Jiang (62) found that individuals who evaluated cancer-related health content negatively were more likely to ignore or avoid it. Moreover, Foust and Taber (63) demonstrated that attitude has a negative effect on information avoidance behavior. Arghashi and Yuksel (64) illustrated a positive relationship between consumers' attitudes toward AR applications and their engagement behaviors. Thus, following the cognitive-affective-behavioral model, perceived usefulness influences attitude, which in turn affects HIA behavior. Based on these findings, the following hypotheses are proposed:

Hypotheses 4 (H4) Offspring's perceived usefulness of the information positively influences their attitude toward parents' health information-sharing behavior.
Hypotheses 5 (H5) Offspring's perceived usefulness of the information negatively influences their HIA behavior.

### 3.4 Relationships between attitude, perceived usefulness, and HIA behavior

In behavioral science, a mediating variable transmits the effect of an independent variable to a dependent variable, thereby clarifying the internal psychological mechanism between them (65). Within the Information Acceptance Model (IAM) and related behavioral frameworks, attitude has been widely recognized as a key mediator between perceived usefulness and behavioral outcomes (66). This mediating effect reflects how individuals' evaluations of usefulness shape their emotional and motivational readiness to act. For instance, Atinafu et al. (67) found that in the context of prenatal mental health care, women's attitudes partially mediated the relationship between perceived usefulness of mobile support tools and their intention to use them. Jum'a et al. (68) further confirmed this mechanism in the context of blockchain use in supply chain management. Foust and Taber (63) also demonstrated that attitudes have a significant negative impact on information avoidance, suggesting that favorable attitudes decrease the likelihood of avoiding health messages. In family-based health communication, offspring may cognitively acknowledge the usefulness of information shared by parents, but their emotional response—captured as “attitude”—plays a key role in determining whether they will engage with or avoid the information. This indicates that attitude not only affects behavior directly, but also functions as a mediator between perceived usefulness and HIA. Based on these theoretical and empirical findings, the following hypotheses are proposed:

Hypotheses 6 (H6) Offspring's attitudes negatively influence their HIA behavior.
Hypotheses 7 (H7) Offspring's attitudes mediate the relationship between perceived usefulness and HIA behavior.

### 3.5 Relationships between perceived usefulness, attitude, information quality, source credibility, and HIA behavior

Information quality and source credibility are widely recognized as core factors influencing individuals' information acceptance and avoidance behaviors (26, 35, 69). High-quality information—defined by accuracy, clarity, and relevance—can directly shape users' cognitive evaluation of its usefulness. The quality of information on social media platforms directly enhances users' perceived usefulness of the information, while source credibility indirectly increases perceived usefulness by altering users' attitudes (57). This highlights a dual-path mechanism in which cognition (usefulness) and affect (attitude) interact. As a mediating variable, perceived usefulness plays a crucial role in the pathway by which information quality and source credibility influence HIA behavior. Perceived usefulness not only serves as a cognitive filter for evaluating information, but also initiates affective responses such as acceptance or avoidance. Madli et al. (37) suggested that when individuals perceive information as having high practical value, they are more likely to develop a positive attitude, thereby reducing HIA behavior. This supports the cognitive-affective-behavioral model in which perceived usefulness shapes attitude, which in turn predicts behavioral outcomes. Zhang and Jiang (62) found that the impact of perceived usefulness on information avoidance behavior is further amplified through the mediating variable of attitude. Therefore, the following hypotheses are proposed:

Hypotheses 8 (H8) Perceived usefulness and attitude have a chain mediating effect in the relationship between information quality and offspring's HIA behavior.
Hypotheses 9 (H9) Perceived usefulness and attitude have a chain mediating effect in the relationship between source credibility and offspring's HIA behavior.

**Figure 1:**
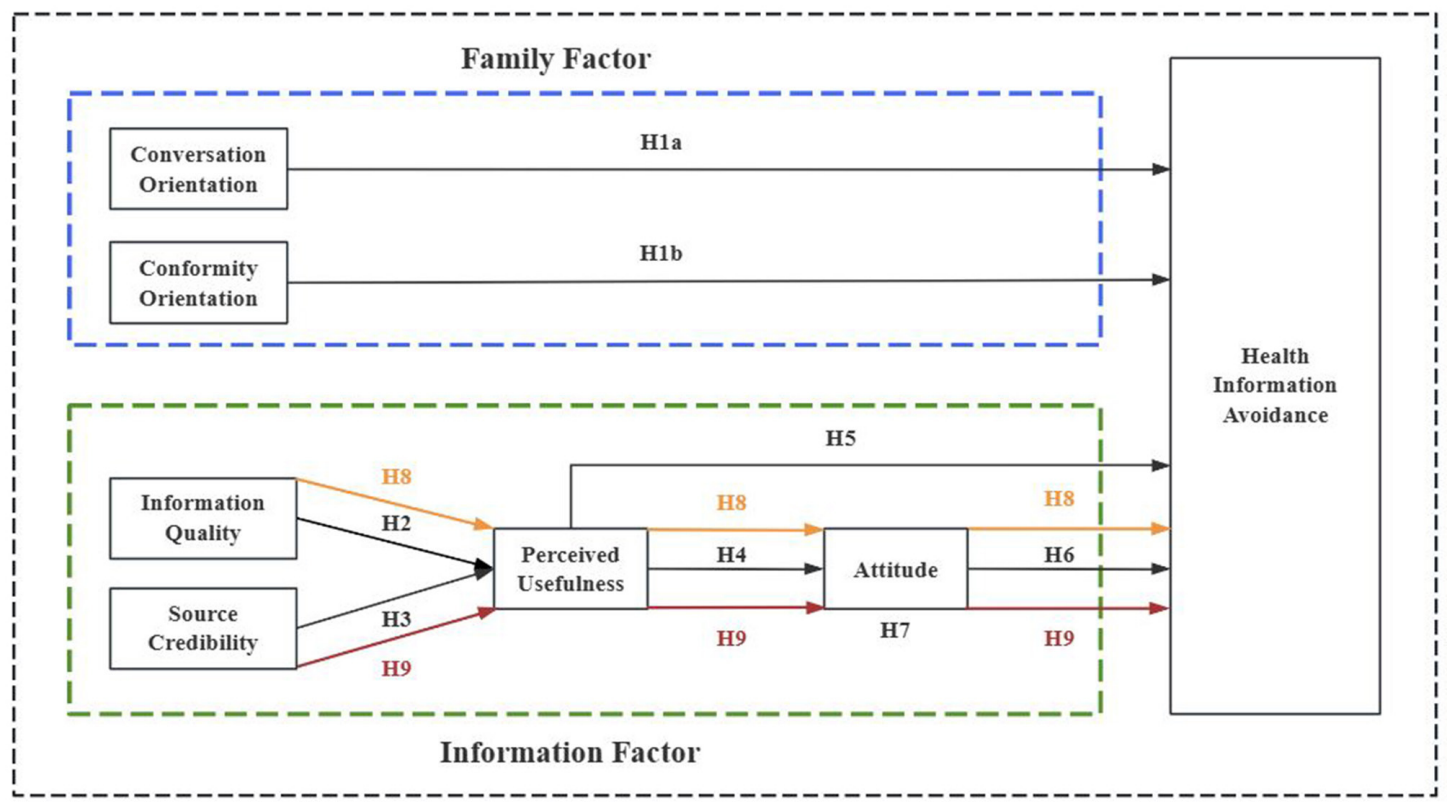
Hypothetical model of the study.

The whole hypothesized model of this study is illustrated in Figure 1.

## 4 Research design

### 4.1 Measurement of variables

The questionnaire consists of three main sections: the first section briefly introduces the purpose and content of this study and obtains informed consent from the respondents. The second section inquires about the respondents' demographic information, such as gender, age, and education level, as well as information about their parents, including age, education level, health status, medical conditions, and lifestyle habits. The third section contains measurement scales for seven variables: information quality, source credibility, perceived usefulness, attitude, HIA behavior, conversation orientation, and conformity orientation.

The measurement scales used in this study were adapted from previously validated instruments. Specifically, items measuring information quality, source credibility, attitude, and health information avoidance behavior were adapted to better align with the research context, with particular emphasis on phrasing relevant to health information. A back-translation procedure was employed to ensure linguistic accuracy and conceptual equivalence across English and Chinese versions. These scales have been tested in prior studies and have demonstrated good reliability and validity, thereby providing a robust foundation for the current research. Additionally, to enhance data quality in a large-scale survey context, one attention check item was embedded in the questionnaire. Respondents were asked a simple arithmetic question (“What is 1 + 3?”), and only those who selected the correct answer were retained for analysis. This approach aimed to filter out inattentive or careless responses caused by fatigue, lack of engagement, or misunderstanding of the survey content. A total of 29 items were included in the final questionnaire (See Appendix 1). All items were measured using a 5-point Likert scale (1 = Strongly Disagree, 5 = Strongly Agree).

- Conversation orientation: five items adopted from Ritchie and Fitzpatrick (30) and Gong et al. (49).
- Conformity orientation: five items adopted from Ritchie and Fitzpatrick (30) and Gong et al. (49).
- Information quality: three items adopted and modified from DeLone and McLean (70).
- Source credibility: three items adopted and revised from Sussman and Siegal (26).
- Perceived usefulness: four items adopted from Hu et al. (71).
- Attitude: four items adapted from Ajzen (72).
- Health information avoidance behavior: five items adopted from Link (73) and Chae et al. (74) measure avoidance behavior.

### 4.2 Data collection and processing

The current study was approved by the Ethics Committee of Guangxi University on January 1, 2024 (Resolution No.GXU-2024-065), as it does not involve biomedical research on humans. Oral informed consent was given by all participants.

The study created the questionnaire through the most popular and professional online survey platform Wenjuanxing (www.wjx.com) in China (75). In order to ensure the scientificity and rigor of the survey, a preliminary survey was conducted before distributing the formal questionnaire. Through the reliability and validity test of 227 pre-survey questionnaires, some items that may have been ambiguous, duplicated other items, or failed to effectively measure the target variable were adjusted and deleted. After these adjustments, another small-scale test of the revised questionnaire was conducted to ensure that all modifications achieved the expected results and to further verify the reliability and validity of the questionnaire. Finally, the formal questionnaires were randomly distributed through social media such as WeChat groups, QQ groups, Weibo, and Douban from January to May 2024.

A total of 1,990 questionnaires were collected. Then, a rigorous data cleaning and preprocessing process were done. In this study, this study applied a minimum response time of 2 s per item (76–78). Given that the questionnaire contains 39 items, any questionnaire completed in <78 s was considered invalid and manually removed from the dataset. Moreover, questionnaires with highly repetitive answer patterns, or failure to pass the attention check were manually excluded to ensure data validity and quality. Finally, a total of 485 invalid questionnaires were eliminated, and 1,505 valid questionnaires were analyzed by SPSS 26.0 and AMOS 28.0.

### 4.3 Reliability and validity tests

Reliability and validity tests were conducted to ensure the quality of the measurement instruments. Cronbach's α was used to assess internal consistency, with all constructs exceeding the recommended threshold of 0.70, indicating good reliability (79). Confirmatory factor analysis (CFA) was performed to evaluate construct validity, including convergent validity and discriminant validity. Convergent validity was confirmed through acceptable values of composite reliability (CR > 0.70) and average variance extracted (AVE > 0.50).

Table 1 shows that the reliability values for the seven constructs are 0.787, 0.742, 0.805, 0.804, 0.846, 0.812, and 0.799, respectively. Each of these values exceeds the threshold of 0.7 (79), indicating that the questionnaire exhibits high reliability and stability. In the assessment of convergent validity, this study follows the recommendations of Hair for validity evaluation (80). Specifically, the absolute value of factor loadings should be at least 0.5, the Average Variance Extracted (AVE) should be above 0.5, and the Composite Reliability (CR) should exceed 0.7, indicating that the questionnaire possesses good convergent validity. Based on the data in Table 1, the factor loadings for each item range between 0.734 and 0.877, all of which are >0.5. The CR values for the seven variables all exceed 0.8, and the AVE values are >0.5. This indicates that the questionnaire has good convergent validity. In addition, in terms of the mean values in Table 1, all constructs fall within a moderate range (~2.95 to 3.32), indicating that the responses are well-distributed without extreme skewness or ceiling/floor effects. This suggests that the scale has good discriminative power. The standard deviations range from 0.80 to 0.97, reflecting moderate variability among respondents. This level of dispersion suggests that the scale captures individual differences effectively, without overly clustered or random responses. Taken together, the distribution and variability of the scale scores support the conclusion that the questionnaire data are valid and of good quality, making them suitable for further statistical analysis.

**Table 1:** Factor loading and confidence levels.

**Construct**	**Items**	**FL**	**Cronbach's α**	**CR**	**AVE**	**Mean (SD)**
IQ	IQ1	0.877	0.787	0.878	0.706	3.08 (0.97)
	IQ2	0.835				
	IQ3	0.808				
SC	SC1	0.808	0.742	0.854	0.661	2.96 (0.88)
	SC2	0.826				
	SC3	0.805				
PU	PU1	0.780	0.805	0.874	0.635	3.02 (0.87)
	PU2	0.806				
	PU3	0.799				
	PU4	0.802				
ATT	ATT1	0.778	0.804	0.872	0.630	3.06 (0.88)
	ATT2	0.814				
	ATT3	0.795				
	ATT4	0.788				
CVO	CVO1	0.777	0.846	0.891	0.620	2.95 (0.88)
	CVO2	0.783				
	CVO3	0.809				
	CVO4	0.779				
	CVO5	0.787				
CFO	CFO1	0.734	0.812	0.870	0.572	3.32 (0.80)
	CFO2	0.750				
	CFO3	0.774				
	CFO4	0.782				
	CFO5	0.739				
HIA	HIA1	0.752	0.799	0.862	0.556	3.28 (0.80)
	HIA2	0.734				
	HIA3	0.740				
	HIA4	0.765				
	HIA5	0.735				

IQ, Information quality; SOC, Source credibility; PU, perceived usefulness; ATT, attitude; CVO, Conversation Orientation; CFO, Conformity Orientation; HIA, Health Information Avoidance; FL, Factor Loadings; CR, Composite Reliability; AVE, Average Variance Extracted; SD, Standard Deviation.

Table 2 presents the means, standard deviations, correlation coefficients, and the square roots of the AVE for the seven variables. This study follows the method proposed by Fornell and Larcker (81), assessing discriminant validity by determining whether the square root of the AVE is greater than the correlation coefficients between the two variables. The square roots of the AVE for the seven variables are 0.840, 0.813, 0.797, 0.794, 0.787, 0.756, and 0.746, all of which are greater than their corresponding correlation coefficients. This indicates that the questionnaire possesses good discriminant validity.

**Table 2:** Correlation coefficient matrix.

**Construct**	**IQ**	**SC**	**PU**	**ATT**	**CVO**	**CFO**	**HIA**
IQ	**0.840**						
SC	0.686	**0.813**					
PU	0.663	0.686	**0.797**				
ATT	0.517	0.516	0.644	**0.794**			
CVO	0.270	0.291	0.372	0.487	**0.787**		
CFO	0.069	0.105	0.070	0.026	−0.238	**0.756**	
HIA	−0.040	0.038	−0.045	−0.120	−0.280	0.494	**0.746**

The bolded diagonal values represent the square root of the Average Variance Extracted (AVE), while the other values represent the correlations between variables.

### 4.4 Common method bias test

Common method bias (CMB) occurs when the covariance between independent and dependent variables is artificially induced due to consistent data sources or raters, identical measurement environments, item context, or the characteristics of the items themselves (82). This artificial covariance between latent variables can lead to misleading research results and conclusions. CMB can undermine the validity of research results and conclusions, making it essential to test for and control this bias in order to ensure the reliability of the study's findings. Therefore, following the recommendation of Zhou and Long (83), this study uses Harman's single-factor test to assess common method bias. In the exploratory factor analysis, all items of the study variables were loaded onto a single factor. The unrotated factor analysis results showed that the variance explained by the first factor was 25.844%, which is below the 40% threshold (84), indicating that there is no significant common method bias in this study.

## 5 Empirical results

### 5.1 Demographics of respondents

Table 3 presents the distribution characteristics of demographic variables for the respondents (offspring) and their parents. The demographic characteristics of the offspring and parental generations exhibit significant differences in several aspects. According to the Seventh National Population Census of China, males account for 51.24% and females for 48.76% of the population. In this study, 44.05% of participants are male and 55.95% are female, a distribution that closely approximates the national gender ratio, indicating good demographic representativeness in terms of gender and providing reference value. In terms of educational attainment, the offspring group exhibits a relatively high level of education, with more than half holding a bachelor's degree or higher (~53.16% in total). This higher educational level likely gives the offspring an advantage in acquiring and processing information, particularly health-related information. Moreover, we limited the study subjects to under 30 years of age: under 18 years (17.34%), 18–25 years (47.38%) and 26–30 years (35.28%). A large proportion of 18–25 year olds, who are basically in college or just entering the workplace, usually have a strong learning ability and a high ability to accept new health information, and they often communicate more closely with their parents. As reported by China Youth Daily (85) in a survey of 1,001 young respondents, 74.6% said that they have placed greater importance on parent-child relationships in recent years and communicate more frequently with their parents.

**Table 3:** Demographics of respondents (*N* = 1,505).

**Variable**	**Characteristics**	**Frequency**	**Percentage (%)**
Gender of offspring	Male	663	44.05
	Female	842	55.95
Education level of offspring	Middle school or below	227	15.08
	High school/technical school/vocational	229	15.22
	Associate degree	249	16.54
	Bachelor's degree	442	29.37
	Graduate degree or above	358	23.79
Age of offspring	Under 18	261	17.34
	18–25	713	47.38
	26–30	531	35.28
Age of parents	Under 40	48	3.19
	40–49	272	18.07
	50–59	599	39.80
	60–69	484	32.16
	70–79	91	6.05
	80 and above	11	0.73
Highest education level of parents	Middle school or below	499	33.16
	High school/technical school/vocational	563	37.41
	Associate degree	158	10.50
	Bachelor's degree	154	10.23
	Graduate degree or above	131	8.70
Residence location of parents	First-tier cities	281	18.67
	Second-tier cities	297	19.73
	Third-tier cities	271	18.01
	Fourth-tier cities or county/town	353	23.46
	Rural	303	20.13
Health status of parents	Poor	341	22.66
	Average	440	29.24
	Good	391	25.98
	Excellent	333	22.13
Health conditions of parents	No illnesses	949	63.06
	Has illnesses	556	36.94
Lifestyle habits of parents	No unhealthy habits	689	45.78
	Has unhealthy habits	816	54.22

In contrast, the age distribution of the parental generation is primarily concentrated in the middle-aged and older adults, with 39.80% aged 50–59 and 32.16% aged 60–69. This indicates that they are in the later stages of life and may be more concerned with health issues and the management of chronic diseases, which can also be reflected by the health status, health conditions and healthy lifestyle habits of their parents. The educational level of them is generally lower than that of the offspring, with the majority having only completed middle school or below (33.16%) and high school/technical school/vocational education (37.41%), which may contribute to their susceptibility to believing incorrect or misleading health information. The parental generation predominantly resides in fourth-tier and below cities or county/town areas (23.46%) and rural areas (20.13%). This indicates that they largely live in regions where medical resources are relatively scarce, which may limit their access to comprehensive healthcare services and up-to-date health information.

### 5.2 Hypothetical research model test

This study employs Structural Equation Modeling (SEM) for hypothesis testing. SEM is a statistical method that analyzes relationships between variables based on the covariance matrix, integrating factor analysis, multiple regression analysis, and path analysis (86). Therefore, using SEM for the empirical research in this study is highly appropriate. The model estimation in SEM is conducted using the Maximum Likelihood method. This study utilizes AMOS 28.0 software to construct the Structural Equation Modeling (SEM). The 1,505 questionnaires collected meet the suggestion of Nunnally and Bernstein that the minimum sample size for SEM analysis should be at least 10 times the number of construct items (87).

#### 5.2.1 Goodness-of-fit test

This study employs the Maximum Likelihood Estimation (MLE) method to evaluate the fit between the proposed model and the observed data (88). MLE is widely used in structural equation modeling (SEM) because it provides efficient, consistent, and unbiased parameter estimates when the data approximate a multivariate normal distribution (89, 90). The goodness-of-fit indices shown in Table 4, such as χ^2^/df, NFI, IFI, TLI, CFI, GFI, and RMSEA, indicate a good fit between the model and the data. Because all the indices meet the required reference values, suggesting that the constructed SEM has a high level of overall fit with the data. Therefore, the model demonstrates good adaptability and can be reliably used for subsequent hypothesis testing.

**Table 4:** Goodness-of-fit indices.

**χ2/df 1 <χ2/df value <3**	**NFI (>0.8)**	**IFI (>0.8)**	**TLI (>0.8)**	**CFI (>0.8)**	**GFI (>0.8)**	**RMSEA (<0.08)**
2.321	0.952	0.972	0.969	0.972	0.963	0.030

#### 5.2.2 Structural equation model texting

The results of the standardized path coefficients for the SEM are shown in Table 5. In terms of family factors, the study found that conversation orientation has a significant negative impact on the offspring's behavior of avoiding health information shared by their parents (β = −0.128, *p* = < 0.001). On the other hand, conformity orientation shows a significant positive impact (β = 0.574, *p* < 0.001), indicating that in this communication style, the offspring are more likely to avoid health information shared by their parents. These findings support the validity of hypotheses H1a and H1b.

**Table 5:** The measurement results of the hypothesized model.

**Hypotheses**	**Paths**	**Standardized coefficient**	**S.E**.	***t*-value**	**Supported**
H1a	CVO → HIA	−0.128^***^	0.033	−3.617	Yes
H1b	CFO → HIA	0.574^***^	0.040	14.806	Yes
H2	IQ → PU	0.227^**^	0.084	2.796	Yes
H3	SC → PU	0.632^***^	0.093	7.285	Yes
H4	PU → ATT	0.734^***^	0.036	19.523	Yes
H5	PU → HIA	−0.049^***^	0.024	−1.901	Yes
H6	ATT → HIA	−0.154^**^	0.061	−2.646	Yes

** *p* < 0.01,

*** *p* < 0.001.

In terms of information factors, the study shows that information quality has a significant positive impact on perceived usefulness (β = 0.227, *p* = 0.005). Additionally, source credibility also has a significant positive impact on perceived usefulness (β = 0.632, *p* < 0.001), indicating that the more the offspring trust the information source, the more likely they are to perceive the information as valuable. These results validate the hypotheses H2 and H3. Further analysis reveals that perceived usefulness has a significant positive impact on attitude (β = 0.734, *p* < 0.001). However, perceived usefulness has a negative impact on the offspring's behavior of avoiding health information shared by their parents (β = −0.049, *p* < 0.001). In addition, attitude also shows a significant negative impact on HIA behavior (β = −0.154, *p* = 0.008 < 0.01). These findings support the validity of hypotheses H4, H5, and H6, and underscore the important role of perceived usefulness and attitude in influencing the offspring's health information avoidance behavior.

The Bootstrap method is statistically more accurate for testing indirect effects compared to the causal steps approach and the product of coefficients approach (91, 92). One of the greatest advantages of the Bootstrap method is that it does not require the indirect effect to follow a normal distribution, unlike the product of coefficients approach (93). Therefore, this study tests the mediation effect using the Bootstrap method within the framework of the SEM. Moreover, the study incorporated demographic variables such as the offspring's gender, age, and education level, as well as the parents' age, education level, and health status, as control variables in the model, which aims to eliminate potential confounding effects from these demographic factors. As shown in Table 6, the study found that the confidence interval for H7 does not include 0 (−0.136, −0.050), indicating that attitude mediates the relationship between perceived usefulness and health information avoidance. The value of the mediation effect is −0.094, supporting the hypothesis H7. In addition, the confidence intervals for both H8 and H9 do not include 0, specifically (−0.069, −0.025) and (−0.087, −0.037), respectively. This indicates that there are chain mediation effects in both H8 and H9, with corresponding mediation effect values of −0.047 and −0.061. Therefore, H8 and H9 are supported.

**Table 6:** Mediation effect results.

**Hypotheses**	**Paths**	**β**	**95% CI**		**p**	**Supported**
			**LLCI**	**ULCI**		
H7	PU → ATT → HIA	−0.094	−0.136	−0.050	0.000	Yes
H8	IQ → PU → ATT → HIA	−0.047	−0.069	−0.025	0.000	Yes
H9	SC → PU → ATT → HIA	−0.061	−0.087	−0.037	0.000	Yes

## 6 Discussion

### 6.1 Family factors and offspring's HIA behavior

The SEM results indicate that conversation orientation negatively impacts offspring's HIA behavior, while a conformity-oriented FCP positively influences it. This finding is consistent with the research of Mullen and Hamilton (94), which suggests that the higher the level of parental control in the parent-child relationship, the more difficult it is for offspring to adopt parental advice and develop viral knowledge (95). Moreover, an important finding is that the absolute value of the standardized coefficient for conformity orientation (0.574) is greater than that for conversation orientation (0.128), indicating that traditional authoritarian communication still holds greater influence in Chinese families. Traditionally, Chinese families place great importance on authority and the hierarchy between elders and younger members. Parents often hold a dominant position within the family, and offspring are generally expected to comply with their parents' wishes without easily opposing or questioning them (3). From a psychological perspective, offspring's avoidance of parental health information may reflect an implicit form of resistance. In conformity-oriented families, where hierarchical expectations are strongly enforced, offspring may lack the space for open negotiation and instead resort to silence or disengagement to protect their autonomy. According to Foucault's theory of discourse and power (96, 97), everyday communication is not neutral, but a medium through which power is exercised and maintained. Health advice, while seemingly benign, can function as a form of discursive control that reinforces parental authority. Therefore, information avoidance may serve as a subtle act of defiance—an attempt by offspring to resist being governed under the guise of care or tradition. This outcome reflects the cultural emphasis on harmony and hierarchy in Chinese families, but also exposes underlying generational tensions rooted in unequal communicative power. As such, HIA behavior should not only be understood as a passive communication failure, but also as an active psychological strategy shaped by relational and cultural dynamics.

Conversation orientation has a negative impact on offspring's HIA behavior. Although its effect is relatively modest, it suggests that offspring in families with more open and egalitarian communication structures are more likely to engage in meaningful dialogue with their parents. As offspring increasingly grow up in environments that emphasize respect, mutual understanding, and emotional support, they tend to feel safer expressing their views and are less inclined to avoid health-related discussions (98). In such conversation-oriented families, offspring are more willing to participate in health information exchanges because they believe their perspectives will be acknowledged and valued.

The results show that although conversation orientation helps reduce offspring's HIA, its influence is still weaker than that of conformity orientation. This may reflect the tension Chinese families face in navigating a shift from hierarchical, authority-driven communication—rooted in Confucian traditions—to more egalitarian and dialogic forms of parent-child interaction. While more families are beginning to value openness and emotional expression, authoritarian styles that emphasize parental dominance and filial obedience remain deeply embedded in the cultural and relational fabric (98). As a result, many families are in a transitional state where coexisting and sometimes conflicting communication norms may hinder the effectiveness of health information exchange.

From a macro-level perspective, this finding has important implications for improving health information dissemination within Chinese families. Health authorities and public health institutions should consider how traditional conformity-based family communication styles may hinder intergenerational understanding. When designing health education strategies, special attention should be paid to addressing hierarchical communication norms that suppress open discussion. This supports calls from previous scholars [e.g., (99)]—to localize health policy frameworks to better accommodate culturally embedded communication patterns.

At the micro-level, this study further suggests that families—particularly parents—should be encouraged to adopt more conversation-oriented communication approaches. In doing so, offspring are more likely to feel psychologically safe, heard, and engaged in health-related dialogue, which reduces avoidance behaviors. This aligns with findings from Hovick et al. (48), who emphasized the role of interpersonal openness in family health communication. However, our study expands this literature by confirming that even when openness is encouraged, the influence of conformity orientation remains stronger in Chinese cultural contexts, underscoring the complex interaction between modern communication ideals and traditional family values.

### 6.2 Information factors and offspring's HIA behavior

Information factors play a crucial role in influencing offspring's HIA behavior. The SEM results indicate that: first, the quality of health information shared by parents and the credibility of the information source both have a positive impact on offspring's perceived usefulness. However, the standardized coefficient for source credibility (0.632) is greater than that for information quality (0.227), indicating that in intergenerational communication, source credibility has a stronger influence on offspring's perception of the usefulness of the information. This finding is consistent with the research of Petty and Cacioppo (100), Hovick et al. (101), and Nelson and Kim (36). This is primarily because, in China, parents typically play an authoritative role in education, and the family environment tends to be one-way instruction rather than two-way communication (102).

In this growing environment, the offspring's ability to make independent decisions and think independently is inhibited to a certain extent, so they are more likely to be influenced and persuaded by external authorities. When they receive health information, they place greater importance on the credibility of information sources, even if the quality of information is not optimal. Secondly, there is a significant association between offspring's perceived usefulness, attitude, and HIA behavior, with attitude serving as a mediator in the relationship between perceived usefulness and HIA behavior. While perceived usefulness plays a crucial role in shaping the initial attitude of offspring toward the information, it is not the sole factor that determines whether they decide to avoid the information. One possible explanation is that in Chinese families, offspring's attitudes toward their parents often include a mix of respect for parental authority, compliance with family expectations, and consideration for family harmony (3). Therefore, even if offspring find the information useful, they may still choose to avoid it if their attitude toward their parents is not positive enough. In addition, family relationships emphasize harmony, respect, and collectivism in Chinese culture (103). Offspring's attitudes toward their parents not only reflects their evaluation of health information itself but also mirrors their perception of family relationships and their identification with the parental role.

The standardized coefficient of attitude is 0.154, which is significantly larger than that of perceived usefulness (0.049). This indicates that in the context of Chinese families, offspring's attitudes have a greater impact on their HIA behavior than the quality and credibility of the information itself. This may be driven by the desire for personal independence of the offspring or by existing conflicts between generations. Therefore, in the context of health communication of Chinese families, it is more important not only to ensure that the health information shared is of high quality and targeted, but also to strive to cultivate mutual understanding and trust among family members, and to enhance offspring's positive attitudes toward their parents. In this way, a more open and supportive family communication climate can be established, thereby encouraging offspring to be more willing to accept and consider the health information provided by their parents.

Finally, information quality and source credibility have a chain effect on offspring's HIA behavior through perceived usefulness and attitude. This effect not only demonstrates the basic laws of information dissemination, but also deeply reveals the particularity of intergenerational communication and health information transmission under the influence of Chinese culture and family values. In Chinese family culture, the dissemination of health information is not just a simple transfer of data; it also carries the deep meaning of trust, respect and emotional interaction among family members (14, 104). The quality of information and the credibility of the source affect offspring's attitude toward information by shaping their perception of the usefulness. This suggests that the attitude is not only a direct response to the content of the information, but also a reflection of offspring's cognition of their parents' roles and family relationships. This reflects the cultural depth of the information dissemination process, in which the transmission of information is not only a logical coherence, but also a form of emotional expression and social.

## 7 Conclusions, limitations and future study

### 7.1 Conclusions

This study, based on FCPs and the AIM theory, comprehensively examined the factors that affect offspring's avoidance of health information shared by their parents. The study finds that at the family level, conversation orientation had a negative impact on offspring's HIA behavior, while conformity orientation had a positive impact on it. This indicates that communication within the family plays a crucial role in the reception and dissemination of health information. At the information level, perceived usefulness and attitude each have a significant negative impact on offspring's HIA behavior. This indicates that perceived usefulness and attitude are key psychological factors that influence offspring's avoidance of health information. In addition, information quality and source credibility have a chain mediating effect on offspring's HIA behavior through perceived usefulness and attitude. This chain mediating effect emphasizes the synergistic interaction of multiple factors in information dissemination, indicating that improving information quality and enhancing the credibility of information sources can effectively increase offspring's acceptance of health information, thereby reducing HIA behavior.

This study's contribution to theory is mainly reflected in two aspects: first, by incorporating both cognitive and relational variables into the framework, this study contributes to existing theory by bridging the IAM and FCPs models in a non-Western setting. It also clarifies how psychological resistance—expressed as health information avoidance—may be shaped by intergenerational discourse power structures. Second, this study extends the application of the IAM framework to the context of family health communication in China. It specifically examines how offspring process health information shared by their parents. This approach not only enriches existing theories of family health communication but also provides new insights into the psychological mechanisms behind intergenerational information transmission and avoidance. Third, the study offers practical implications for public health interventions and digital health design. Health campaigns targeting younger audiences should not only focus on improving the quality and credibility of content but also consider the relational context in which health messages are received.

### 7.2 Limitations and future study

Although this study provides valuable findings and insights, there are still some limitations. Firstly, this study employed SEM to analyze and validate various factors and their interrelationships. The advantage of SEM lies in its ability to handle complex variable relationships, providing a comprehensive validation of the research model. However, the model falls short in explaining certain key phenomena. For instance, while the model accounted for a significant portion of the variance in offspring's HIA, it did not fully capture the emotional or contextual factors (105, 106)—such as parent-child relational closeness or digital literacy—that may also influence avoidance behavior. These omitted variables could offer additional explanatory power and deserve further investigation in future research. Moreover, the use of two existing scales to measure FCPs and HIA, though validated in prior research, may not fully capture the complexity or cultural specificity of these constructs in Chinese families. Some culturally nuanced aspects of communication—such as implicit obedience or indirect resistance (107, 108)—may fall outside the scope of standardized instruments. Future research could benefit from using mixed methods or culturally adapted scales to enhance construct validity.

Secondly, although the age limit for the study was under 30 years old, the age span was still large. Therefore, future research could further examine the heterogeneity of this model across different age groups to more precisely identify the unique factors influencing health information avoidance among offspring. On the basis of comparative studies, a more explanatory model of the mechanisms behind HIA in offspring could be constructed, leading to the development of more targeted health intervention strategies for this group.

Finally, although the gender distribution of the sample (male: 44.05%, female: 55.95%) deviates from national census data, this discrepancy primarily affects the representativeness of the sample and may limit the generalizability of the findings. However, since the focus of this study lies in examining the relational patterns among key variables rather than producing population-level estimates, the impact of this gender imbalance on the core analytical logic is minimal. Future research may consider more balanced sampling to enhance external validity.

## Data Availability

The datasets presented in this study can be found in online repositories. The names of the repository/repositories and accession number(s) can be found in the article/supplementary material.

## Appendix

**Table A1:** Summary of questionnaire scale items.

**Constructs**	**Items**		**Cronbach's α**
IQ	IQ1	The health information my parents share with me is useful and relevant to my health concerns.	0.787
	IQ2	The health information my parents share with me is comprehensive.	
	IQ3	The health information my parents share with me is accurate.	
SOC	SOC1	The sources of the health information my parents share are reliable.	0.742
	SOC2	The sources of the health information my parents share are trustworthy.	
	SOC3	The sources of the health information my parents share are authoritative.	
PU	PU1	I believe following the health information in the messages is helpful.	0.805
	PU2	I believe following the health information in the messages is beneficial to me.	
	PU3	I believe following the health information in the messages is useful to me.	
	PU4	Overall, I believe following the health information in the messages is a good thing to do.	
ATT	ATT1	Receiving health information shared by my parents is valuable to me.	0.804
	ATT2	Receiving health information from my parents makes me feel happy.	
	ATT3	I enjoy sharing health information with my parents.	
	ATT4	Overall, I have a positive attitude toward receiving health information from my parents.	
CVO	COV1	My parents and I often share our thoughts with each other.	0.846
	COV2	My parents and I often talk about our feelings and emotions.	
	COV3	I really enjoy chatting with my parents.	
	COV4	My parents and I often talk about our plans and hopes for the future.	
	COV5	My parents and I frequently share details of our daily lives.	
CFO	CON1	My parents expect me to obey them without question.	0.812
	CON2	In my family, my parents usually have the final say.	
	CON3	My parents get upset when I express different opinions.	
	CON4	At home, I am expected to follow the rules set by my parents.	
	CON5	My parents often say things like “You'll understand when you grow up.	
HIA	HB1	I avoid discussing health information with my parents online.	0.799
	HB2	Sometimes, I ignore the health information shared by my parents.	
	HB3	I would rather not know the health information provided by my parents.	
	HB4	I generally do not follow the health information or advice provided by my parents (such as health products, knowledge, beliefs, or behaviors).	
	HB5	I always avoid talking to others about the health information provided by my parents.	

## References

[1] Daines CL Hansen D Novilla MLB Crandall A. Effects of positive and negative childhood experiences on adult family health. BMC Public Health. (2021) 21:1–8. 10.1186/s12889-021-10732-w 33820532 PMC8022401

[2] Hurst JL Widman L Maheux AJ Evans-Paulson R Brasileiro J Lipsey N. Parent–child communication and adolescent sexual decision making: an application of family communication patterns theory. J Fam Psychol. (2022) 36:449. 10.1037/fam0000916 34472938

[3] Gao F Zhang J. Raising a filial child: how health information concerns and family communication patterns influence people sharing health information with parents. Chin J Journal Commun. (2023) 12:117–35. 10.13495/j.cnki.cjjc.2023.12.007

[4] Chen J Gan L. Information-seeking relationships: a study on the health information sharing behavior of the elderly on WeChat. Journal Rev. (2021) 9, 10–24. 10.16057/j.cnki.31-1171/g2.2021.09.003

[5] Zhang W Fuligni AJ. Authority, autonomy, and family relationships among adolescents in urban and rural China. J Res Adolesc. (2006) 16:527–37. 10.1111/j.1532-7795.2006.00506.x

[6] Zhou T Li Y Wang X Li S. Study on the current status and influencing factors of health information avoidance among college students. Libr Sci Res. (2023) 5:70–78+69. 10.15941/j.cnki.issn1001-0424.2023.05.008

[7] Eckhardt G. Culture's consequences: comparing values, behaviors, institutions and organisations across nations. Aust J Manag. (2002) 27:89–94. 10.1177/031289620202700105

[8] Sweeny K Melnyk D Miller W Shepperd JA. Information avoidance: Who, what, when, and why. Rev Gen Psychol. (2010) 14:340–53. 10.1037/a0021288

[9] Duan Q Zhang D Xie X Wang S. Silent “resistance”: a study on information avoidance behavior among digital natives—taking forwarded online information from parents as the research focus. Libr Tribune. (2023) 43:26–38.

[10] Ketcham R. The Idea of Democracy in the Modern Era. Lawrence, KS: University Press of Kansas (2021).

[11] Lin L Wang Q. Adolescents' filial piety attitudes in relation to their perceived parenting styles: an urban–rural comparative longitudinal study in China. Front Psychol. (2022) 12:750751. 10.3389/fpsyg.2021.750751 35140649 PMC8818790

[12] Born VDL Vasbø KB. “Doing authority”: stories of parental authority across three generations. J Marriage Fam. 87:114–33. 10.1111/jomf.13028

[13] Tian J Huang H. Family communication model theory: a theoretical aspect of family communication. Youth Journal. (2023) 8:38–40. 10.15997/j.cnki.qnjz.2023.08.040

[14] Gong W Guo Q Jiang L. The feeding effect in health transmission: the influence of intergenerational communication on infectious disease prevention and control behavior in middle-aged and elderly people. J Zhejiang Univ. (2021) 51:42–53.

[15] Yao X Shen B. Disembedding and dissociation in mediatization of family communication: an investigation of intergenerational communication on a vaccine information WeChat platform. Journal Evol. (2024) 1:76–85.

[16] Peng L Zhang Q. Research on influencing factors and correlation pathways of health information avoidance behavior in middle-aged and elderly people: an exploratory analysis based on grounded theory. Res Libr Sci. (2022) 1:77–86+76. 10.15941/j.cnki.issn1001-0424.2022.01.011

[17] Wu X. Research on influencing factors of WeChat users' health information sharing behavior. Chin J Journal Commun. (2022) 10:96–118.34900917

[18] Li H Wen Y. Study on influencing factors of online health information sharing willingness based on meta-analysis. Data Anal Knowl Discov. (2023) 12:125–41.

[19] Shan Y Huang S. Relationships between intergenerational support and the health information avoidance behavior of the elderly. Front Soc Sci Technol. (2021) 3:88–92. 10.25236/FSST.2021.03011422724914

[20] Peng L Tang L Li Q. Study on the key influencing factors of health information avoidance behavior of the elderly under public health emergencies. Sci Inf Res. (2024) 2:100–14.

[21] Gong W Ouyang X. Cognitive bias and communication dilemmas: Intergenerational health information communication in the elderly. J Southwest Minzu Univ. (2021) 6:192–8.

[22] Howell JL Shepperd JA. Reducing information avoidance through affirmation. Psychol Sci. (2012) 23:141–5. 10.1177/0956797611424164 22241812

[23] Ho DYF. “Filial piety and its psychological consequences.” In: Bond MH, editors. The handbook of Chinese psychology. Oxford, UK: Oxford University Press (1996). p. 155–65.

[24] Yeh KH Bedford O. A test of the dual filial piety model. Asian J Soc Psychol. (2003) 6:215–28. 10.1046/j.1467-839X.2003.00122.x

[25] Yan Y. The impact of traditional filial piety on Chinese people. Psychoanal Inq. (2025) 1–9. 10.1080/07351690.2025.2461593

[26] Sussman SW Siegal WS. Informational influence in organizations: an integrated approach to knowledge adoption. Inf Syst Res. (2003) 14:47–65. 10.1287/isre.14.1.47.1476719642375

[27] Wang Y. Information adoption model, a review of the literature. J Econ Bus Manag. (2016) 4:618–22. 10.18178/joebm.2016.4.11.462

[28] Elwalda A Erkan I Rahman M Zeren D. Understanding mobile users' information adoption behaviour: an extension of the information adoption model. J Enterp Inf Manag. (2022) 35:1789–811. 10.1108/JEIM-04-2020-012928347450

[29] Feng Y. For myself or for others? The influence of family communication patterns on family health history communication and online health information seeking. J Health Commun. (2025) 30:72–81. 10.1080/10810730.2025.2450617 39804582

[30] Ritchie LD Fitzpatrick MA. Family communication patterns: measuring intrapersonal perceptions of interpersonal relationships. Communic Res. (1990) 17:523–44. 10.1177/009365090017004007

[31] Koerner AF Fitzpatrick MA. Toward a theory of family communication. Commun Theory. (2002) 12:70–91. 10.1111/j.1468-2885.2002.tb00260.x

[32] McKnight DH Kacmar CJ. “Factors and effects of information credibility.” In: *Proceedings of the Ninth International Conference on Electronic Commerce*. New York, NY: Association for Computing Machinery (2007). p. 423–32. 10.1145/1282100.1282180

[33] Hilligoss B Rieh SY. Developing a unifying framework of credibility assessment: construct, heuristics, and interaction in context. Inf Process Manag. (2008) 44:1467–84. 10.1016/j.ipm.2007.10.001

[34] Park DY Kim H. Determinants of intentions to use digital mental healthcare content among university students, faculty, staff: motivation, perceived usefulness, perceived ease of use, and parasocial interaction with AI Chatbot. Sustainability. (2023) 15:872. 10.3390/su15010872

[35] Hussain S Ahmed W Jafar RMS Rabnawaz A Jianzhou Y. eWOM source credibility, perceived risk and food product customer's information adoption. Comput Human Behav. (2017) 66:96–102. 10.1016/j.chb.2016.09.034

[36] Nelson JL Kim SJ. Improve trust, increase loyalty? Analyzing the relationship between news credibility and consumption. Journalism Pract. (2021) 15:348–65. 10.1080/17512786.2020.1719874

[37] Madli F Sondoh S Totu A Janin Y Syed Annuar SN Cham TH. Modelling organ donation information adoption among Malaysian youth using the information adoption model (IAM). Int J Pharm Healthc Mark. (2024) 18:252–75. 10.1108/IJPHM-08-2022-0077

[38] Meng M You J Liu C Zeng Z. Health information adoption behavior research: concept definition, theoretical model, future prospects. Mod Inform. (2024) 44:157–67. 10.3969/j.issn.1008-0821.2024.06.013

[39] Ma X Chen L. Seeking online health information for aged parents in China: a multigroup comparison of the comprehensive model of information seeking based on eHealth literacy levels. Int J Commun. (2023) 17:2326–47. Available online at: https://ijoc.org/index.php/ijoc/article/view/19891/4112

[40] Ajzen I. The theory of planned behavior. Organ Behav Hum Decis Process. (1991) 50:179–211. 10.1016/0749-5978(91)90020-T

[41] Park T. How information acceptance model predicts customer loyalty? A study from perspective of eWOM information. Bottom Line. (2020) 33:60–73. 10.1108/BL-10-2019-0116

[42] Le XC. Determinants of health information acceptance to COVID-19 avoidance: The lens of information acceptance model and elaboration likelihood model. Bottom Line. (2023) 36:29–51. 10.1108/BL-04-2021-0058

[43] McLeod JM Chaffee SR. “The construction of social reality.” In: Tedeschi JT, editor. The Social Influence Processes. New York: Routledge. p. 50–99. 10.4324/9781315134970-2

[44] Rauscher EA Schrodt P Campbell-Salome G Freytag J. The intergenerational transmission of family communication patterns: (In)consistencies in conversation and conformity orientations across two generations of family. J Fam Commun. (2020) 20:97–113. 10.1080/15267431.2019.1683563

[45] Pabundu DD Ramadhana MR. Role of family communication and boarding school system in forming child independence. Mediator J Komunikasi. (2023) 16:303–20. 10.29313/mediator.v16i2.2710

[46] Schrodt P Witt PL Messersmith AS. A meta-analytical review of family communication patterns and their associations with information processing, behavioral, psychosocial outcomes. Commun Monogr. (2008) 75:248–69. 10.1080/03637750802256318

[47] Hesse C Rauscher EA. The relationship between family communication patterns and child vaccination intentions. Commun Res Rep. (2016) 33:61–7. 10.1080/08824096.2015.1117444

[48] Hovick SR Thomas SN Watts J Tan NQP. The influence of family communication patterns on the processing of messages to increase family health history seeking intentions. Health Commun. (2019) 36:424–32. 10.1080/10410236.2019.1693129 31749383

[49] Gong W Jiang LC Guo Q Shen F. The role of family communication patterns in intergenerational COVID-19 discussions and preventive behaviors: a social cognitive approach. BMC Psychology. (2023) 11:290. 10.1186/s40359-023-01331-y 37752573 PMC10523603

[50] Zhang Q. Family communication patterns and conflict styles in Chinese parent-child relationships. Commun Q. (2007) 55:113–28. 10.1080/01463370600998681

[51] Kang SY Lee JA Kim YS. Impact of family communication on self-rated health of couples who visited primary care physicians: A cross-sectional analysis of Family Cohort Study in Primary Care. PLoS ONE. (2019) 14:e0213427. 10.1371/journal.pone.0213427 30865692 PMC6415836

[52] Ye R Wu Y Sun C Wang Q Mao Y Raat H. Health communication patterns and adherence to a micronutrient home fortification program in rural China. J Nutr Educ Behav. (2022) 54:36–45. 10.1016/j.jneb.2021.07.014 34690077

[53] Xie QW Chen R Wang K Lu J Wang F Zhou X. Associations of latent patterns of parent–child communication with communication quality and mental health outcomes among Chinese left-behind children. BMC Public Health. (2024) 24:332. 10.1186/s12889-024-17793-7 38297309 PMC10829291

[54] Scheinfeld E. The role of shame, stigma, and family communication patterns in the decision to disclose STIs to parents in order to seek support. Int J Environ Res Public Health. (2023) 20:4742. 10.3390/ijerph20064742 36981650 PMC10048974

[55] Moss CE Waddell TF Thomas S. Hesitancy in the home: the relationship between family communication patterns and willingness to converse about COVID-19 vaccination. Am Behav Scientist. (2022). 10.1177/00027642221146118

[56] Bakhtiari A Kashefi F Pasha H Nasiri-Amiri F Bakouei F Saffari E. Role of family communication patterns as predictors of behavioral health among students of public universities in north of Iran. J Educ Health Promot. (2024) 13:120. 10.4103/jehp.jehp_1406_23 38726074 PMC11081464

[57] Komendantova N Zobeidi T Yazdanpanah M. How do instagram messages affect the use of renewable energy?–application of an extended information adoption model. J Environ Informatics. (2024) 43:129–40. 10.3808/jei.202400515

[58] Liu J Kong J. Why do users of online mental health communities get likes and reposts: a combination of text mining and empirical analysis. Healthcare. (2021) 9:1133. 10.3390/healthcare9091133 34574907 PMC8470014

[59] Zhou T. Understanding online health community users' information adoption intention: an elaboration likelihood model perspective. Online Inform Rev. (2022) 46:134–46. 10.1108/OIR-09-2020-0412

[60] Peng Y Yin P Deng Z Wang R. Patient–physician interaction and trust in online health community: the role of perceived usefulness of health information and services. Int J Environ Res Public Health. (2020) 17:139. 10.3390/ijerph17010139 31878145 PMC6981828

[61] Tan PSH Seow AN Choong YO Tan CH Lam SY Choong CK. University students' perceived service quality and attitude towards hybrid learning: ease of use and usefulness as mediators. J Appl Res High Educ. (2024) 16:1500–14. 10.1108/JARHE-03-2023-0113

[62] Zhang L Jiang S. Examining the role of information behavior in linking cancer risk perception and cancer worry to cancer fatalism in China: cross-sectional survey study. J Med Internet Res. (2024) 26:e49383. 10.2196/49383 38819919 PMC11179024

[63] Foust JL Taber JM. Information avoidance: past perspectives and future directions. Perspect Psychol Sci. (2025) 20:241–63. 10.1177/17456916231197668 37819241

[64] Arghashi V Yuksel CA. Interactivity, inspiration, and perceived usefulness! how retailers' AR-apps improve consumer engagement through flow. J Retailing Consumer Serv. (2022) 64:102756. 10.1016/j.jretconser.2021.102756

[65] Zhou M Zhao L Kong N Campy KS Qu S Wang S. Factors influencing behavior intentions to telehealth by Chinese elderly: an extended TAM model. Int J Med Informatics. (2019) 126:118–27. 10.1016/j.ijmedinf.2019.04.001 31029253

[66] Gajanayake R Sahama T Iannella R. “The role of perceived usefulness and attitude on electronic health record acceptance.” In: *2013 IEEE 15th Int. Conf. on e-Health Networking, Applications and Services (Healthcom 2013)*. IEEE (2013). p. 388–93. 10.1109/HealthCom.2013.6720706

[67] Atinafu WT Tilahun KN Yilma TM Mekonnen ZA Walle AD Adem JB. Intention to use a mobile phone to receive mental health support and its predicting factors among women attending antenatal care at public health facilities in Ambo town, West Shoa zone, Ethiopia 2022. BMC Health Serv Res. (2023) 23:1368. 10.1186/s12913-023-10392-z 38057856 PMC10701993

[68] Jum'a L Mansour M Zimon D Madzík P. A two-model integrated technology adoption framework for using blockchain in supply chain management: attitude towards blockchain as a mediator. J Sci Technol Policy Manage. (2024). 10.1108/JSTPM-05-2023-0068

[69] Schäfer S Betakova D Lecheler S. Zooming in on topics: an investigation of the prevalence and motives for selective news avoidance. Journalism Stud. (2024) 25:1423–40. 10.1080/1461670X.2024.2338114

[70] DeLone WH McLean ER. The DeLone and McLean model of information systems success: a ten-year update. J Manag Inf Syst. (2003) 19:9–30. 10.1080/07421222.2003.11045748

[71] Hu PJ Chau PY Sheng ORL Tam KY. Examining the technology acceptance model using physician acceptance of telemedicine technology. J Manag Inf Syst. (1999) 16:91–112. 10.1080/07421222.1999.11518247

[72] Ajzen I. Constructing a TPB Questionnaire: Conceptual and Methodological Considerations. University of Massachusetts Amherst, Office of Information Technologies (2002). Available online at: https://citeseerx.ist.psu.edu/document?repid=rep1&type=pdf&doi=0574b20bd58130dd5a961f1a2db10fd1fcbae95d

[73] Link E. Information avoidance during health crises: Predictors of avoiding information about the COVID-19 pandemic among German news consumers. Inf Process Manag. (2021) 58:102714. 10.1016/j.ipm.2021.102714 34539039 PMC8441302

[74] Chae J Lee CJ Kim K. Prevalence, predictors, and psychosocial mechanism of cancer information avoidance: findings from a national survey of US adults. Health Commun. (2020) 35:322–30. 10.1080/10410236.2018.1563028 30606065

[75] Wang Z Tang Y Cui Y Guan H Cui X Liu Y. Delay in seeking health care from community residents during a time with low prevalence of COVID-19: A cross-sectional national survey in China. Front Public Health. (2023) 11:1100715. 10.3389/fpubh.2023.1100715 36895687 PMC9989024

[76] Curran PG. Methods for the detection of carelessly invalid responses in survey data. J Exp Soc Psychol. (2016) 66:4–19. 10.1016/j.jesp.2015.07.006

[77] Soland J Wise SL Gao L. Identifying disengaged survey responses: new evidence using response time metadata. Appl Meas Educ. (2019) 32:151–65. 10.1080/08957347.2019.1577244

[78] Zhong X Li M Li L. Control and identification of careless responses in questionnaire surveys. Adv Psychol Sci. (2021) 29:225–37. 10.3724/SP.J.1042.2021.0022537113526

[79] DeVellis RF. Scale Development: Theory and Applications. New York: Sage Publications (1991).

[80] Hair JF Black WC Babin BJ Anderson RE. Multivariate Data Analysis (7th ed.). Prentice Hall, Upper Saddle River, NJ (2010).

[81] Fornell C Larcker DF. Evaluating structural equation models with unobservable variables and measurement error. J Mark Res. (1981) 18:39–50. 10.1177/002224378101800104

[82] Podsakoff PM MacKenzie SB Lee JY Podsakoff NP. Common method biases in behavioral research: a critical review of the literature and recommended remedies. J Appl Psychol. (2003) 88:879. 10.1037/0021-9010.88.5.879 14516251

[83] Zhou H Long L. Statistical test and control method of common method deviation. Adv Psychol Sci. (2004) 6:942–50.

[84] Tang D Wen Z. Common method deviation testing: problems and recommendations. J Psychol Sci. (2020) 1:215–23. 10.16719/j.cnki.1671-6981.20200130

[85] China Youth Daily. 74.6% of Surveyed Youth Believe People are Paying More Attention to Parent-child Relationships. (2024). Available online at: https://www.xinhuanet.com/local/20241031/4b521ae8d50644f1b02477b638b1a473/c.html (accessed April 21, 2025).

[86] Maydeu-Olivares A. Maximum likelihood estimation of structural equation models for continuous data: standard errors and goodness of fit. Struct Equ Modeling. (2017) 24:383–94. 10.1080/10705511.2016.1269606

[87] Nunnally JC Bernstein IH. Psychometric Theory. New York: McGraw-Hill (1994).

[88] Hair JF Sarstedt M Ringle CM Mena JA. An assessment of the use of partial least squares structural equation modeling in marketing research. J Acad Mark Sci. (2012) 40:414–33. 10.1007/s11747-011-0261-6

[89] Byrne BM. Structural Equation Modeling with Mplus: Basic Concepts, Applications, and Programming. New York, NY: Routledge (2013). 10.4324/9780203807644

[90] Kline RB. Principles and Practice of Structural Equation Modeling. Guilford Publications (2023).

[91] MacKinnon DP Lockwood CM Williams J. Confidence limits for the indirect effect: Distribution of the product and resampling methods. Multivariate Behav Res. (2004) 39:99–128. 10.1207/s15327906mbr3901_4 20157642 PMC2821115

[92] Williams J MacKinnon DP. Resampling and distribution of the product methods for testing indirect effects in complex models. Struct Equ Modeling. (2008) 15:23–51. 10.1080/10705510701758166 20179778 PMC2825896

[93] Davison AC Hinkley DV. Bootstrap Methods and their Application. United Kingdom: Cambridge University Press (1997). 10.1017/CBO9780511802843

[94] Mullen C Hamilton NF. Adolescents' response to parental Facebook friend requests: the comparative influence of privacy management, parent-child relational quality, attitude, peer influence. Comput Human Behav. (2016) 60:165–72. 10.1016/j.chb.2016.02.026

[95] Egbert N Zhu Y Choi M Beam MA Smith TC. Family communication patterns and parents' intentions to vaccinate their child against COVID-19. Health Commun. (2023) 38:2774–81. 10.1080/10410236.2022.2114768 36017868

[96] Foucault M. Discipline and Punish: The Birth of the Prison. New York: Vintage Books (1977).

[97] Miller S. Foucault on discourse and power. Theoria. (1990) 76:115–25.

[98] Zhu X. Bridging the knowledge gap: international frontiers and independent construction of home communication research. J Soc Sci Hunan Norm Univ. (2023) 3:123–33. 10.19503/j.cnki.1000-2529.2023.03.014

[99] Chen J Huang C. “Self-oriented” or “family-oriented”? A study on the effectiveness of influenza vaccination communication strategies among Chinese college students. Int Journal. (2020) 42, 98–113. 10.13495/j.cnki.cjjc.2020.06.006

[100] Petty RE Cacioppo JT. Communication and Persuasion: Central and Peripheral Routes to Attitude Change. New York: Springer Verlag (1986). 10.1007/978-1-4612-4964-1

[101] Hovick SR Kahlor L Liang MC. Personal cancer knowledge and information seeking through PRISM: the planned risk information seeking model. J Health Commun. (2014) 19:511–27. 10.1080/10810730.2013.821556 24433251 PMC8582150

[102] Guo Y. Educational approaches in the VUCA era: parenting strategies of authoritative parents. Popul Psychol. (2024) 12:32–4.

[103] Tian SM. The unique temperament of family communication in China and its value in the contemporary era. Educ Media Res. (2023) 4:79–84.

[104] Wu R. Emotional participation: a practical approach to cross-cultural transmission of cultural meaning: a case study of the spread of “grassland complex” in mixed-language families in Inner Mongolia. J Commun Rev. (2024) 77:1–8.

[105] Long H Ma S. The relationship between experiential avoidance, cognitive fusion, and mental health among college students: the mediating role of mindfulness. Chin J Health Psychol. (2021) 29:1077–84.

[106] Wang Y Tian J Yang Q. Experiential avoidance process model: a review of the mechanism for the generation and maintenance of avoidance behavior. Psychiatr Clin Psychopharmacol. (2024) 34:179. 10.5152/pcp.2024.23777 39165887 PMC11332439

[107] Gao G Kao K Ting-Toomey S. Communicating Effectively with the Chinese (Vol. 5). Thousand Oaks, CA: Sage Publications (1998). 10.4135/9781452220659

[108] Chien CL. Beyond authoritarian personality: the culture-inclusive theory of Chinese authoritarian orientation. Front Psychol. (2016) 7:924. 10.3389/fpsyg.2016.00924 27445894 PMC4927584

